# Endoscopic Resection of a Pedunculated Brunner's Gland Hamartoma of the Duodenum

**DOI:** 10.1155/2016/6707235

**Published:** 2016-08-08

**Authors:** Masaya Iwamuro, Takehiro Tanaka, Satoko Ando, Tatsuhiro Gotoda, Hiromitsu Kanzaki, Seiji Kawano, Yoshiro Kawahara, Hiroyuki Okada

**Affiliations:** ^1^Department of Gastroenterology and Hepatology, Okayama University Graduate School of Medicine, Dentistry, and Pharmaceutical Sciences, 2-5-1 Shikata-cho, Kita-ku, Okayama 700-8558, Japan; ^2^Department of General Medicine, Okayama University Graduate School of Medicine, Dentistry, and Pharmaceutical Sciences, 2-5-1 Shikata-cho, Kita-ku, Okayama 700-8558, Japan; ^3^Department of Pathology, Okayama University Hospital, Okayama 700-8558, Japan; ^4^Department of Endoscopy, Okayama University Hospital, Okayama 700-8558, Japan

## Abstract

A 68-year-old Japanese woman presented with a solitary pedunculated polyp in the duodenum. Endoscopic ultrasonography showed multiple cystic structures in the polyp. The polyp was successfully resected by endoscopic snare polypectomy and pathologically diagnosed as Brunner's gland hamartoma. Because hamartomatous components were not identified in the stalk of the polyp, we speculate that the stalk developed from traction of the normal duodenal mucosa. When a solitary, pedunculated polyp with cystic structure within the submucosa is found in the duodenum, Brunner's gland hamartoma should be considered in the differential diagnosis, despite the rarity of the disease. This case underscores the usefulness of endoscopic ultrasonography for the diagnosis of duodenal subepithelial tumors.

## 1. Introduction

Brunner's glands, also called duodenal glands, are found in the submucosa of the duodenum, typically in the duodenal bulb and in the second portion of the duodenum proximal to the sphincter of Oddi. Brunner's glands produce a mucus-rich, bicarbonate-containing, alkaline secretion that aids in neutralizing the acidic content of chyme and gastric acid, providing an alkaline milieu to optimize intestinal absorption and lubricate the intestinal walls [1]. Lesions associated with Brunner's gland vary from hyperplasia, hamartoma, and, in rare instances, adenocarcinoma [1–3].

We recently encountered a patient with a pedunculated polyp in the second portion of the duodenum, which was covered with intact duodenal mucosa. The polyp was successfully resected by endoscopic snare polypectomy. The diagnosis of Brunner's gland hamartoma was based on pathological analysis of the resected specimen. In this report, we speculate the mechanism of morphogenesis of this pedunculated feature, mainly focusing on the pathological characteristics. Moreover, we discuss differential diagnosis of duodenal lesions showing submucosal origin.

## 2. Case Report

**Figure 1:**
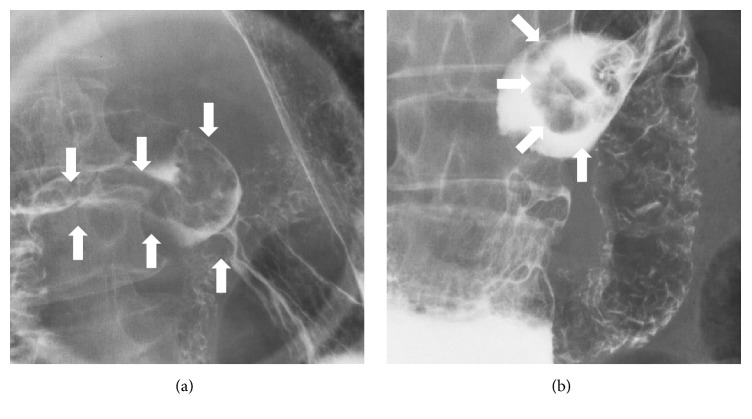
Barium follow-through examination images. A pedunculated polyp can be identified in the duodenum (arrows).

**Figure 2:**
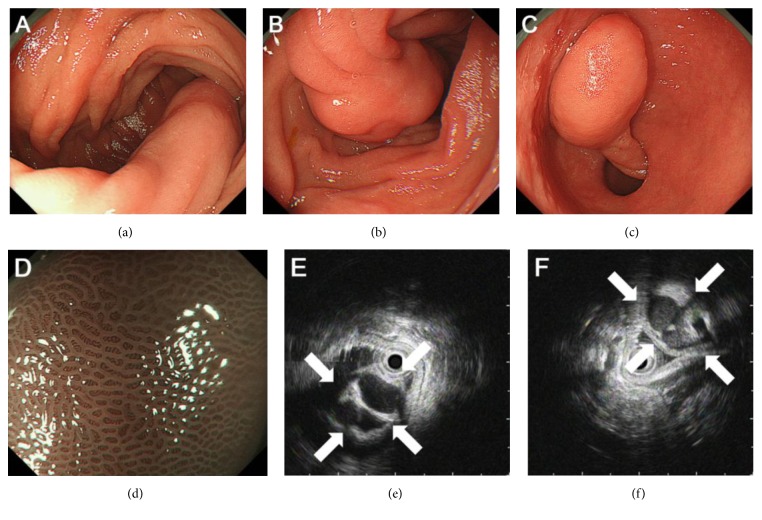
Endoscopy images. Esophagogastroduodenoscopy results showed a pedunculated polyp measuring approximately 20 mm ((a), (b)). The polyp was easily extended to the stomach by traction with biopsy forceps (c). Magnifying observation with narrowband imaging revealed that the surface of the polyp was entirely covered with intact duodenal mucosa (d). Endoscopic ultrasonography showed multiple cystic structures ((e), (f), arrows).

A 68-year-old Japanese woman underwent barium follow-through examination for the upper gastrointestinal tract as part of a routine medical screening. A pedunculated polyp was detected in the duodenum (Figure 1). The patient was referred to our hospital for further investigation and treatment of the polyp. The patient had been taking medication for hyperlipidemia but had no history of gastrointestinal diseases. A physical examination revealed no abnormalities, and there was no evidence of peripheral lymphadenopathy. Laboratory findings revealed an elevated level of total cholesterol to 254 mg/dL, but levels of carcinoembryonic antigen and carbohydrate antigen 19-9 and blood cell counts were within normal ranges. Esophagogastroduodenoscopy showed a pedunculated polyp in the duodenal second portion. The head of the polyp was approximately 20 mm in size (Figures 2(a) and 2(b)). There were no erosions or ulcers in the polyp. The tumor had long stalk; therefore it easily migrated to the stomach (Figure 2(c)). Magnifying observation with narrowband imaging confirmed that the surface was entirely covered with intact duodenal mucosa, suggesting a subepithelial origin of the tumor (Figure 2(d)). Endoscopic ultrasonography visualized at least three cystic structures in the polyp head (Figures 2(e) and 2(f)). Based on the macroscopic and ultrasonography features, Brunner's gland hamartoma was highly suspected.

**Figure 3:**
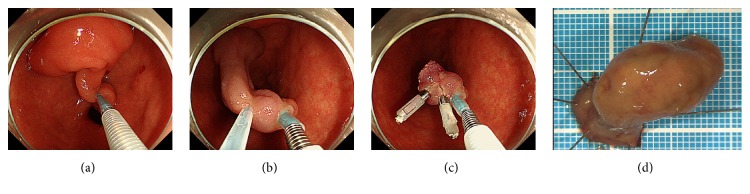
Endoscopic resection of the polyp. During the procedure, the stalk was strangulated with a detachable snare (a). The polyp was then resected by snare polypectomy (b). The cut surface was closed with two metal clips (c). The size of the resected specimen was 26 × 19 mm (d).

**Figure 4:**
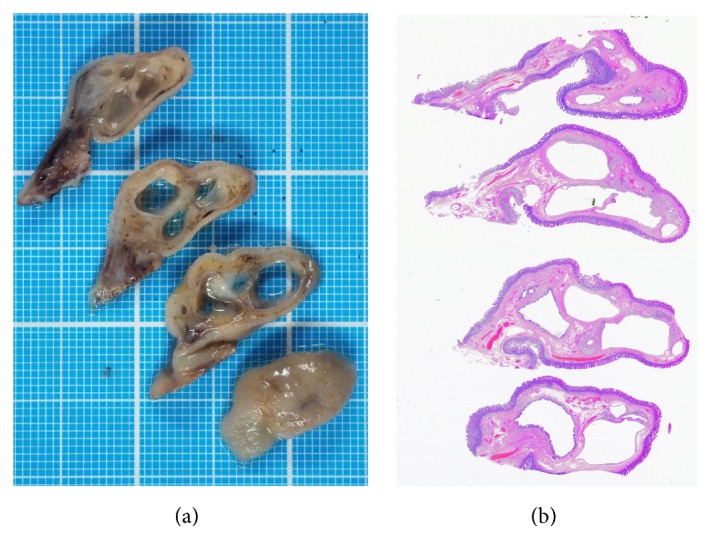
Images of the resected specimen. Macroscopically, multiple cystic structures can be observed in the head of the polyp, whereas no cyst is visible in the stalk.

**Figure 5:**
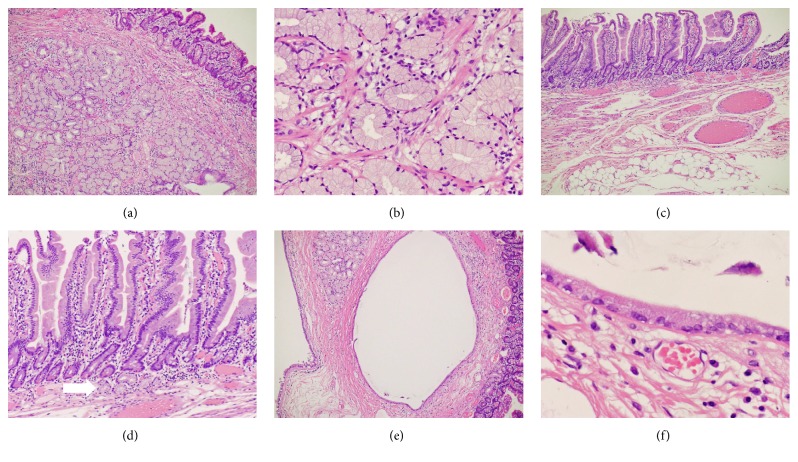
Pathological images. Stromal cell proliferation and lobular arrangement of proliferated Brunner's glands separated by fibromuscular septa are visible in the head of the polyp ((a), ×4; (b), ×20). In the stalk, multiple blood vessels and adipose tissues can be seen ((c), ×4; (d), ×10). Brunner's glands are only partly visible in the stalk ((d), arrow). The cystic structure visible in the head of the polyp is lined with columnar cells ((e), ×4; (f), ×40). Based on the pathological features, a diagnosis of Brunner's gland hamartoma was reached.

The patient agreed to undergo endoscopic resection of the polyp to prevent duodenal obstruction due to impaction of the tumor. First, we repositioned the polyp from the duodenum to the stomach by traction with biopsy forceps. The stalk was then strangulated with a detachable snare (Figure 3(a)). Next, the polyp was successfully resected using snare polypectomy (Figure 3(b)). Although there was no bleeding from the resected section, the cut surface was closed with two metal clips (Figure 3(c)). The size of the resected specimen was 26 × 19 mm (Figure 3(d)). Macroscopically, multiple cystic structures were observed in the head of the polyp, whereas no cyst was detected in the stalk (Figure 4). Pathological evaluation revealed a lobular arrangement of proliferated Brunner's glands separated by fibromuscular septa at the head of the polyp (Figures 5(a) and 5(b)). Stromal cell proliferation was also noted. In contrast, Brunner's glands were only partly identified in the stalk (Figures 5(c) and 5(d)). Multiple blood vessels and abundant adipose tissue were observed in the stalk. The cystic structure present in the head of the polyp was lined with columnar cells without atypia, indicating that the cysts consisted of dilated ducts of Brunner's glands (Figures 5(e) and 5(f)).

## 3. Discussion

Brunner's gland hyperplasia and hamartoma are two representative lesions that arise from Brunner's gland. Although distinct definitions do not exist that discriminate the two lesions, generally hyperplasia indicates multiple lesions less than 1 cm, whereas hamartoma refers to a solitary lesion larger than 1 cm [2, 3]. Macroscopically, Brunner's gland hyperplasia appears as multiple, sessile, submucosal nodules mostly identified in the duodenal bulb and/or in the second portion of the duodenum. Brunner's gland hamartoma typically appears as a polypoid, pedunculated lesion ranging in size from 0.7 to 12 cm, with a mean of 4 cm [1]. Pathologically, Brunner's gland hyperplasia is characterized by an excessive number of Brunner's glands separated by fibrous septa. Brunner's gland hamartoma consists of Brunner's glands, ducts, smooth muscle, adipose tissue, and lymphoid cells [4–6]. In the present patient, Brunner's gland hamartoma appeared with the typical morphology, presenting as a solitary pedunculated polypoid mass with a stalk consisting of normal duodenal mucosa [3, 6, 7]. Dislocation of the polyp head from the duodenum to the gastric lumen has also been described previously [8].

Patients with Brunner's gland hamartoma may be asymptomatic and the lesion is usually discovered incidentally [9]. Possible symptoms caused by Brunner's gland hamartoma include duodenal obstruction, intussusception, obstructive jaundice, pancreatitis, and bleeding [1, 3, 4]. Thus, resection is indicated for symptomatic or large lesions to relieve or prevent complications [10]. Successful treatment has been reported using endoscopic resection techniques such as snare polypectomy and endoscopic mucosal resection [11–15].

Pathological evaluation of Brunner's gland hamartoma in the present patient revealed that the polyp head consisted of proliferated Brunner's glands in lobules, stromal cells, and marked cystic dilatation lined by columnar cells (Figures 5(a), 5(b), 5(e), and 5(f)). In contrast, such hamartomatous components were not present in the stalk (Figures 5(c) and 5(d)). Consequently, we speculate that peristalsis of the duodenum acted as a traction force on the hamartoma. The mucosa and submucosa were then extended, resulting in the final formation of the stalk. Because the hamartoma was localized at the head of the polyp, the complete resection of the lesion could easily be performed by cutting the stalk using an endoscopic snare polypectomy procedure. In our case, we considered that when performing endoscopic resection of Brunner's gland hamartoma, prevention of hemorrhage or hemostasis was important, since multiple blood vessels were identified in the stalk in our patient (Figure 5(c)). We used a detachable snare before resection of the polyp and it was quite useful to prevent bleeding from the cut surface (Figure 3).

Various types of subepithelial tumors occur in the duodenum, varying from Brunner's gland hyperplasia, Brunner's gland hamartoma, neuroendocrine tumors, gastrointestinal stromal tumors, leiomyomas, ectopic pancreas, lipomas, gangliocytic paragangliomas, lymphomas, and varices [3]. Among these, Brunner's gland hamartomas, lipomas, and gangliocytic paragangliomas can be pedunculated. Lipomas appear as a soft, round, yellowish mass covered with a smooth surface. Endoscopic ultrasonography shows a homogenous, hyperechoic mass in the submucosal layer. Gangliocytic paragangliomas are rare tumors characterized by triphasic cellular differentiation composed of epithelioid neuroendocrine cells, spindle cells with Schwann cell differentiation, and ganglion cells [16–18]. The tumor presents as a single lesion in the duodenum exhibiting a polypoid, pedunculated, or sessile tumor [19]. On endoscopic ultrasonography, gangliocytic paragangliomas are typically seen as an isoechoic mass in the submucosal layer [20]. Hizawa et al. reviewed the endoscopic ultrasonography features of six cases of Brunner's gland hamartoma and they noted that single or multiple cystic structures within the submucosa could be detected in four patients and solid echogenicity in the remaining two patients [10, 21]. Although the cystic structure may be found in the duodenum in association with Brunner's gland hyperplasia, duplication cyst, ectopic pancreas, central necrosis of gastrointestinal stromal tumor, pancreatic pseudocyst, and varices, these lesions do not present a pedunculated appearance. Therefore, endoscopic ultrasonography provides endoscopists with an important tool with which to diagnose pedunculated lesions in the duodenum.

In conclusion, we treated a patient with Brunner's gland hamartoma showing pedunculated morphology. Hamartomatous components were observed in the head of the polyp, suggesting that the stalk resulted from the traction of the normal duodenal mucosa. When a solitary, pedunculated polyp with cystic structure is identified at the duodenum, it is important for clinicians to consider Brunner's gland hamartoma in the differential diagnosis, despite the rarity of the disease.
